# Physics and Application of Superconductivity

**DOI:** 10.3390/ma19122574

**Published:** 2026-06-15

**Authors:** Paola Romano

**Affiliations:** 1 Department of Sciences and Technologies, Sannio University, 82100 Benevento, Benevento, Italy; promano@unisannio.it; 2 Consiglio Nazionale delle Ricerche CNR-SPIN, UOS Salerno, 84084 Fisciano, Salerno, Italy

## 1. Introduction and Scope

The phenomenon of superconductivity was first observed in 1911 [1], and since then, a wide variety of materials have been found to exhibit this remarkable property under specific conditions. In particular, superconductivity emerges below a characteristic temperature, known as the critical temperature (Tc), which uniquely identifies each material. For several decades, superconductivity was limited to very low temperatures, typically close to that of liquid helium, making its practical application challenging [2].

A major breakthrough occurred in 1987 with the discovery of high-temperature superconductivity in layered cuprates by Bednorz and Müller, who observed a superconducting transition in LaBa_2_CuO_4−x_ [3]. Shortly afterward, superconductivity above the liquid nitrogen temperature was achieved in YBa_2_Cu_3_O_x_, opening up new possibilities for technological applications [4]. Since then, extensive experimental and theoretical efforts have led to the discovery and investigation of numerous classes of superconductors, including cuprates, iron-based superconductors, and other unconventional systems [5].

The Special Issue “*Physics and Applications of Superconductivity*” aims to provide an overview of recent developments in the field, spanning both fundamental and applied aspects. Contributions address different classes of superconducting materials—such as low-temperature, high-temperature, and iron-based systems—focusing on their structural, electrical, and magnetic properties, as well as on theoretical modeling. In addition, attention is devoted to material processing, fabrication techniques, and design strategies required to optimize performance and enable practical applications.

## 2. An Overview of the Published Article

The first edition of this Special Issue includes eight research papers and one review paper.

Recent advances in superconductivity and energy materials continue to reveal how subtle microscopic mechanisms can produce unexpected macroscopic phenomena, while simultaneously opening new technological perspectives. In this context, Galluzzi et al. report anomalous magnetic features in the quasi-one-dimensional compound K_2_Cr_3_As_3_, including temperature-independent spike-like magnetization jumps around ~800 Oe. These results, attributed to frustration-driven instabilities arising from the twisted triangular lattice, suggest that the system lies close to a ferrimagnetic phase [6]. This highlights the delicate balance between competing interactions and the role of fluctuations in low-dimensional superconductors.

A complementary advance in superconducting materials engineering is presented by Gajda et al., who demonstrate that high isostatic pressure annealing can induce a highly textured nanofiber structure in MgB_2_ wires, with characteristic dimensions reduced to the nanoscale. This microstructural control leads to a significant enhancement of critical current density, directly linked to an increased density of effective pinning centers [7,8]. These findings show that thin-film-like performance can be achieved in scalable bulk architectures, representing an important step toward practical high-field applications.

Talantsev revisits the issue of the apparent violation of the Pauli limiting field in magic-angle multilayer graphene. By incorporating strong coupling effects into the theoretical framework, the study reconciles experimental observations with conventional superconducting theory [9,10]. This reinterpretation removes the need to invoke unconventional pairing mechanisms and provides a unified description of critical field behavior in moiré superconductors.

In their work, Soomro et al. highlight a critical limitation in the evaluation of AC losses in high-temperature superconductors, demonstrating that rotating magnetic fields can significantly increase dissipation compared to one-dimensional excitations. Their combined experimental and numerical analysis underscores the importance of considering realistic electromagnetic conditions when designing superconducting devices, particularly for rotating machinery and power applications.

The central role of materials synthesis is emphasized in the review by Hou et al. [11], where different crystal growth techniques for 11 iron-based superconductors are systematically compared. The study shows how precise control of synthesis conditions is essential to access intrinsic electronic properties and to resolve complex phenomena such as nematicity, multiband effects, and quantum criticality. This establishes a direct link between materials processing and the fundamental physics governing superconductivity.

Several contributions demonstrate how superconducting materials can be used for interesting applications. Scarpa et al. show that the FeSe superconductor can act as an efficient supercapacitor electrode, achieving high capacitance and long-term stability, competitive with state-of-the-art transition metal selenides [12]. Similarly, Scarpa et al. report that the intermetallic compound CeCu_2_Si_2_ can be engineered into a porous microstructured electrode with excellent electrochemical performance, where the synergistic redox activity of Ce and Cu plays a key role [13]. These works illustrate the growing convergence between superconductivity and energy storage research.

At the interface between superconductivity and fundamental physics, Grimaudo et al. propose a Josephson junction-based platform for axion detection. By introducing a quasipotential framework, the study shows that axion coupling reduces the effective energy barrier, thereby modifying the escape dynamics of the junction. This effect leads to measurable changes in switching statistics, providing a promising route for detecting dark matter candidates using superconducting devices [14].

Finally, Nigro et al. [15] investigate the superconducting gap structure of electron-doped cuprates Nd_2−x_Ce_x_CuO_4−δ_ using point-contact spectroscopy on bulk polycrystalline samples. The observation of a robust gap feature (~5 meV), together with the absence of a zero-bias conductance peak, suggests deviations from a simple d-wave symmetry and supports more complex pairing scenarios [16]. High-bias conductance further reveals the role of barrier properties and inelastic processes [17], confirming the effectiveness of this technique in probing anisotropic superconducting states.

Taken together, these studies illustrate a coherent picture in which advances in synthesis, characterization, and theoretical modeling converge to deepen our understanding of superconductivity while expanding its application domain. From frustrated magnetism to quantum sensing and energy storage, superconducting materials are increasingly emerging as versatile platforms at the nexus of condensed matter physics and advanced technologies.

## 3. Conclusions

The contributions presented in this Special Issue highlight the breadth and vitality of current research in superconductivity, spanning from fundamental physics to emerging technological applications. Several studies provide new insights into complex physical phenomena, such as magnetic instabilities in low-dimensional systems, unconventional superconducting behavior, and the interplay between competing electronic orders. Meanwhile, significant progress has also been achieved in materials engineering, demonstrating how microstructural control and advanced synthesis techniques can directly enhance superconducting performance.

Overall, this Special Issue emphasizes the strong connection between material design, experimental characterization, and theoretical modeling in advancing the field. The results presented here not only deepen the understanding of superconducting systems but also pave the way for future developments in both fundamental science and practical applications.

## Data Availability

Not applicable.
